# Efficacy of Nucleotide/Nucleoside Analogues and Hepatitis B Immunoglobulin Therapy in Blocking Mother-to-Child Transmission of Hepatitis B in an Eastern Chinese Group

**DOI:** 10.1155/2020/4305950

**Published:** 2020-12-17

**Authors:** Xiaojun Sun, Chengwei Wang, Bian Wang, Xiuzhen Yang, Hongtao Xu, Meilong Shen, Kuichun Zhu

**Affiliations:** ^1^Taizhou Hospital of Chinese Traditional Medicine, Multidisciplinary Center, Taizhou, Jiangsu 225300, China; ^2^Taizhou People's Hospital, Department of Hepatology, Taizhou, Jiangsu 225300, China; ^3^Taizhou Hospital of Chinese Traditional Medicine, Department of Gastroenterology & Hepatology, Taizhou, Jiangsu 225300, China; ^4^Wuxi Shenrui Bio-Pharmaceuticals Co., Ltd., R&D Department, Wuxi, Jiangsu 214000, China

## Abstract

The objective of this study was to investigate the efficacy and potential side-effects of nucleotide/nucleoside analogues and hepatitis B immunoglobulin injection of newborns in blocking mother-to-child transmission of hepatitis B virus in the middle and late pregnancy period. 238 cases of enrolled pregnant women were divided into the Telbivudine group, the Tenofovir group, the Lamivudine group, and the hepatitis B immunoglobulin (HBIG) group. Enrolled patients received corresponding therapies. Clinical and laboratory data were collected. Results showed that the levels of HBV DNA of the enrolled pregnant women in the Telbivudine, Tenofovir, and Lamivudine groups decreased rapidly after 12 weeks of drug intervention compared with those in the control. HBsAg positive rate in newborns and in children 24 weeks after birth was 0/60, 0/60, 0/60, 3/30, and 11/28 in the Telbivudine, Tenofovir, Lamivudine, HBIG, and control groups, respectively. No significant side-effects were identified after following up to 12 months after birth. Our results show that routine HBV vaccine plus HBIG injections is insufficient in blocking mother-to-child HBV transmission. Administration of nucleotide/nucleoside analogues or HBIG at pregnancy is suggested to maximize the blocking of vertical HBV transmission.

## 1. Introduction

The hepatitis B virus (HBV) infection remains a major health problem in China and worldwide. Infant infection from pregnant women as hepatitis B carriers is still a major concern [1–3]. For mothers with HBV infection in China, newborns are injected with HBIG and hepatitis B vaccine to interrupt mother-to-child transmission of HBV. However, mother-to-child transmission of HBV still accounts for about 30% to 50% new HBV infection [4]. Researches had shown that mother-to-child transmission of HBV mainly occurred in late pregnancy and lactation [3, 5, 6]. nucleotide/nucleoside analogues (NA) have been used in addition to HBIG and HBV vaccine to interrupt vertical transmission of HBV [7]. To search for more effective approaches in blocking the vertical transmission, we treated pregnant women in middle and late pregnancy period with nucleotide/nucleoside analogues and treated newborns with HBIG to interrupt mother-to-child transmission of HBV, and our results added more thoughts to the prevention strategies of mother-to-child transmission.

## 2. Materials and Methods

### 2.1. Subjects, Grouping, and Treatment

With the approval of the Ethics Committee of Taizhou People's Hospital, all the enrolled pregnant women signed the informed consent form. All 238 pregnant women were chronic HBV carriers who received obstetric examination in Taizhou People's Hospital from September 2010 to April 2018. Diagnosis was made according to the diagnostic criteria established in 2010 in China [8]. No participant received interferon or NA treatment before the study. The blood HBV titers of the enrolled pregnant women were all greater than 10^4^ IU/ml. Patients with primary liver cancer, other viral hepatitis, fatty hepatitis, alcoholic hepatitis, drug hepatitis, and autoimmune hepatitis were excluded.

After enrollment, patients were divided into the Telbivudine group, the Tenofovir group, the Lamivudine group, the hepatitis B immunoglobulin (HBIG) group, and the control group randomly. The age, height, and body weight of pregnant women in each group were comparable. The details of treatment for each group of pregnant women are shown in Table 1. Alanine aminotransferase, total bilirubin, HBsAg, HBeAg, and HBV DNA were routinely tested after enrollment and reexamined every 12 weeks. At the 12th gestational week, HBV genotyping and drug resistance were examined. The percentage and absolute value of natural killer (NK) cells in the blood at 12 gestational weeks were also examined.

Newborns were routinely injected with hepatitis B vaccine on the day of birth, and HBIG 200 IU was injected at 2 weeks, 4 weeks, and 12 weeks, respectively. Hepatitis B markers were tested using the cord blood of the newborn and the blood at the age of 24 weeks. Pregnant women and newborns were followed up for at least 1 year after childbirth.

### 2.2. Laboratory Tests

HBV DNA level was tested by the 7300 real-time qPCR instrument and kit of the American Applied Biosystems company according to the manufacturer's instructions. The Abbott AxSYM reagent and instrument were used as the automatic immunoassay system. The titer of HBsAg was expressed as *S*/*N*, that is, the ratio of the sample to the negative control. It was considered positive when *S*/*N* ≥ 2. The result of HBeAg was determined by the ratio of sample value to clinical cutoff value, and it was considered positive when the ratio ≥ 1. The percentage of total NK cells (CD3-CD16+56+) in lymphocytes was examined with flow cytometry, and the absolute number of NK cells was calculated based on blood white blood cell counts. HBV genotyping and drug resistance mutations were performed with PCR amplification of HBV polymerase region and DNA sequencing with an ABl3100 sequencing instrument. All laboratory tests were performed with nationally certified test methods and materials.

### 2.3. Statistical Analysis

The rates between groups were analyzed by the *χ*^2^ test, and the quantitative measurement data were analyzed by a two-sample *t*-test. Statistical analysis was carried out by the SPSS16.0 software. *P* < 0.05 was considered as statistically significant.

## 3. Results

The levels of HBV DNA of the enrolled pregnant women in the Telbivudine, Tenofovir, and Lamivudine groups decreased rapidly after 12 weeks of NA treatment, compared with those in the control group, *P* < 0.05 (Table 2). The decrease in HBV levels in the HBIG group was limited (Table 2). There were 2/60, 1/60, 1/60, 4/30, and 12/28 HBsAg-positive newborn cases in the Telbivudine, Tenofovir, Lamivudine, HBIG, and control groups, respectively, at birth. Reexamined at 24 weeks after birth, there were 0/60, 0/60, 0/60, 3/30, and 11/28 newborn cases of HBsAg positivity in the Telbivudine, Tenofovir, Lamivudine, HBIG, and control groups, respectively, *P* < 0.05 (Table 3).

There was no severe adverse reaction during the treatment period in the treatment groups. Transient mild increase in serum creatine kinase was found in 1 case in the Telbivudine group and 1 case in the Lamivudine group; the level returned to normal after two weeks. There was no significant difference in gestational age, mean weight, and Apgar score among all groups (Table S1). There were no complications such as fetal distress, neonatal asphyxia, amniotic fluid contamination, abnormal development, or malformations in each group. When the infants in each group were followed up to the age of 12 months, no deformity or other abnormalities were found.

All the patients enrolled in this study carried HBV genotype B or C. Our results showed that HBsAg, HBeAg, genotyping, HBV drug resistance, or the number of NK cells was not significantly correlated with treatment outcomes in patients (Table S2).

## 4. Discussion

Mother-to-child transmission is still a main transmission route of HBV infection in China. Over 90% of the newborns who get infected HBV develop into chronic hepatitis B carrier status [9, 10]. Infants infected by mother-to-child transmission could carry HBV for dozens of years [4]. High HBV DNA level and abnormal liver function lead to high mother-to-child transmission rates and adverse pregnancy events [11]. At present, active and passive immune intervention still could not completely interrupt the transmission from mother to child, with about 9% newborns from HBV carrier mothers still getting infected [12]; the infection rate could be around 40% if no intervention is provided [13].

High HBV DNA level can easily lead to placental dysplasia which makes the trophoblast cells of the placenta lose the protective barrier function, resulting in transplacental hematogenous and cell-derived intrauterine infection [4, 7]. Therefore, reducing HBV DNA level is considered the key to restore the placental barrier and reduce intrauterine transmission of HBV [14, 15]. Recent studies had shown that Telbivudine, Tenofovir, and Lamivudine could effectively reduce the HBV DNA level in pregnant women [7, 16]. Our study showed that HBV DNA titers of pregnant women in the Telbivudine, Tenofovir, and Lamivudine groups were significantly decreased after 12 weeks of treatment, consistent with the literature [17]. At 24 weeks after birth, the HBsAg positive rate was 0/60, 0/60, 0/60, 3/30, and 11/28 in the Telbivudine, Tenofovir, Lamivudine, HBIG, and control groups, respectively. The significant differences indicate that the interruption approaches at pregnancy were effective in blocking HBV transmission. HBIG is a concentrated anti-HBV passive immune protection agent [18]. HBIG can activate the complement system, enhance humoral immunity, and significantly reduce the HBV load in pregnant women [19]. Our results showed that 3 of the 30 newborns in the HBIG group tested HBsAg positive at 24 weeks after birth. Compared with the HBIG group, the Telbivudine, Tenofovir, and Lamivudine groups showed stronger inhibition of HBV. Genotype, drug resistance, absolute value of NK cells, HBsAg, and HBeAg in pregnant women with hepatitis B carriers did not correlate with treatment outcomes.

Early pregnancy is the most important period of infant development growth, so the safety of Telbivudine, Tenofovir, and Lamivudine was of great concern [17, 20]. Telbivudine and Tenofovir are class B drugs for pregnancy, and Lamivudine is a class C drug for pregnancy. Consistent with the literature reports, our results showed that Telbivudine, Tenofovir, and Lamivudine were relatively safe [21, 22].

In summary, our results showed that administration of nucleotide/nucleoside analogues Telbivudine, Tenofovir, and Lamivudine and, to a lesser extent, the sequential hepatitis B immunoglobulin injections in pregnant women were effective in blocking mother-to-child transmission of hepatitis virus. Routine HBV vaccine plus HBIG injections was insufficient in blocking mother-to-child HBV transmission. Administration of nucleotide/nucleoside analogues or HBIG at pregnancy is suggested to maximize the blocking of vertical HBV transmission.

## Figures and Tables

**Table 1:** Study groups and treatments.

Groups	Cases	Treatments
Telbivudine group	60	Telbivudine 600 mg qd, from 26 gestational weeks to 4 weeks postpartum
Tenofovir group	60	Tenofovir 300 mg qd, from 26 gestational weeks to 4 weeks postpartum
Lamivudine group	60	Lamivudine 100 mg qd, from 26 gestational weeks to 4 weeks postpartum
HBIG group	30	HBIG 200 IU/4 weeks for 3 times from 26 gestational weeks
Control group	28	No NA or HBIG intervention before birth

**Table 2:** The blood HBV DNA levels in study subjects.

Group	Before treatment	12 weeks after treatment
Telbivudine group	6.69 ± 1.72	2.18 ± 1.35^#^
Tenofovir group	6.72 ± 1.56	2.26 ± 1.40^#^
Lamivudine group	6.80 ± 1.59	2.34 ± 1.44^#^
HBIG group	6.77 ± 1.51	5.17 ± 1.83
Control group	6.72 ± 1.64	6.80 ± 1.56

Numbers were *M* ± SD of the group at log scale. ^#^Statistical significance compared with the control group, *P* < 0.05.

**Table 3:** HBsAg status in newborns.

Groups	Cases	HBsAg+ cases at birth	HBsAg+ cases 24 weeks after birth
Telbivudine group	60	2	0^#^^∗^
Tenofovir group	60	1	0^#^^∗^
Lamivudine group	60	1	0^#^^∗^
HBIG group	30	4	3^#^
Control group	28	12	11

^#^Statistical significance compared with the control group, *P* < 0.05. ^∗^Statistical significance compared with the HBIG group, *P* < 0.05.

## Data Availability

All data generated or analyzed in this study are included in the article and its supplementary information files.

## Supplementary Materials

Supplementary Materials Table S1: characteristics of newborns. Table S2: other characteristics of subjects in each group.
