# Supplementary material for: Efficacy of Nucleotide/Nucleoside Analogues and Hepatitis B Immunoglobulin Therapy in Blocking Mother-to-Child Transmission of Hepatitis B in an Eastern Chinese Group
Source: Infect Dis Obstet Gynecol. 2020 Dec 17;2020:4305950. doi: 10.1155/2020/4305950 (PMC7759418; doi:10.1155/2020/4305950)
Supplement: Supplementary Materials — Table S1: characteristics of newborns. Table S2: other characteristics of subjects in each group. [file 4305950.f1.docx]

Table S1. Characteristics of Newborns

| group | gestational age | weight | Cesarean rate | Apgar score |
| --- | --- | --- | --- | --- |
| Telbivudine group | 39.41±0.86 | 3.51±0.50 | 8/60 | 9.41±0.46 |
| Lamivudine group | 39.53±0.91 | 3.63±0.51 | 7/60 | 9.40±0.47 |
| Tenofovir group | 39.56±0.91 | 3.64±0.51 | 7/60 | 9.41±0.47 |
| HBIG group | 39.46±0.84 | 3.52±0.67 | 3/30 | 9.38±0.52 |
| control group | 39.57±0.82 | 3.56±0.48 | 3/28 | 9.42±0.52 |

Numbers were M±SD of the group.

Table S2. Other characteristics of subject in each group

| group | HBsAg* | HBeAg* | genotype（B/C) | HBV drug resistance | NK cell count（/ul） |
| --- | --- | --- | --- | --- | --- |
| Telbivudine group | 2076.3±1729.2 | 467.4±213.6 | 24/36 | 0 | 361.1±135.8 |
| Tenofovir group | 1945.6±1870.3 | 471.2±236.7 | 22/38 | 0 | 360.3±147.5 |
| Lamivudine group | 1985.6±1833.8 | 456.2±274.1 | 22/38 | 0 | 352.6±148.3 |
| HBIG group | 2186.3±1726.4 | 470.4±251.3 | 9/21 | 0 | 348.2±145.0 |
| control group | 2085.6±1843.9 | 457.5±264.0 | 8/20 | 0 | 347.0±161.0 |

Numbers were M±SD of the group.

*Those are relative levels (see the Materials and Methods for details)
